# Supplementary material for: Multisectoral actions in primary health care: A realist synthesis of scoping review
Source: PLoS One. 2023 Aug 10;18(8):e0289816. doi: 10.1371/journal.pone.0289816 (PMC10414560; doi:10.1371/journal.pone.0289816)
Supplement: S1 File — (DOCX) [file pone.0289816.s001.docx]

**Supporting Information**

**Table S1: Realist and Meta-narrative Evidence Syntheses: Evolving Standards (RAMESES) publication standards for realist synthesis.**

| **TITLE** | | |  |
| --- | --- | --- | --- |
| 1 |  | In the title, identify the document as a realist synthesis or review | Title, Page 1 |
| ABSTRACT | | |  |
| 2 |  | While acknowledging publication requirements and house style, abstracts should ideally contain brief details of: the study's background, review question or objectives; search strategy; methods of selection, appraisal, analysis and synthesis of sources; main results; and implications for practice. | Abstract, Page 2 |
| INTRODUCTION | | |  |
| 3 | Rationale for review | Explain why the review is needed and what it is likely to contribute to existing understanding of the topic area. | Introduction, Page 4 |
| 4 | Objectives and focus of review | State the objective(s) of the review and/or the review question(s). Define and provide a rationale for the focus of the review. | Introduction, Page 4 |
| METHODS | | |  |
| 5 | Changes in the review process | Any changes made to the review process that was initially planned should be briefly described and justified. | Methods, Page 5 |
| 6 | Rationale for using realist synthesis | Explain why realist synthesis was considered the most appropriate method to use. | Methods, Page 5 |
| 7 | Scoping the literature | Describe and justify the initial process of exploratory scoping of the literature. | Methods, Page 5 |
| 8 | Searching processes | While considering specific requirements of the journal or other publication outlet, state and provide a rationale for how the iterative searching was done. Provide details on all the sources accessed for information in the review. Where searching in electronic databases has taken place, the details should include, for example, name of database, search terms, dates of coverage and date last searched. If individuals familiar with the relevant literature and/or topic area were contacted, indicate how they were identified and selected. | Methods, Pages 5 and 6 |
| 9 | Selection and appraisal of documents | Explain how judgements were made about including and excluding data from documents and justify these. | Methods, Page 6 |
| 10 | Data extraction | Describe and explain which data or information were extracted from the included documents and justify this selection. | Methods, Page 7, and supplementary material |
| 11 | Analysis and synthesis processes | Describe the analysis and synthesis processes in detail. This section should include information on the constructs analyzed and describe the analytic process. | Methods, Page 7 |
| RESULTS | | |  |
| 12 | Document flow diagram | Provide details on the number of documents assessed for eligibility and included in the review with reasons for exclusion at each stage as well as an indication of their source of origin (for example, from searching databases, reference lists and so on). You may consider using the example templates (which are likely to need modification to suit the data) that are provided. | Results, Page 8 |
| 13 | Document characteristics | Provide information on the characteristics of the documents included in the review. | Results, Page 8, and supplementary material |
| 14 | Main findings | Present the key findings with a specific focus on theory building and testing. | Results, Page 8-13 |
| DISCUSSION | | |  |
| 15 | Summary of findings | Summarize the main findings, taking into account the review's objective(s), research question(s), focus and intended audience(s). | Discussion, Page 13-14 |
| 16 | Strengths, limitations and future research directions | Discuss both the strengths of the review and its limitations. These should include (but need not be restricted to) (a) consideration of all the steps in the review process and (b) comment on the overall strength of evidence supporting the explanatory insights which emerged. The limitations identified may point to areas where further work is needed. | Discussion, Page 16 |
| 17 | Comparison with existing literature | Where applicable, compare and contrast the review's findings with the existing literature (for example, other reviews) on the same topic. | Discussion, Page 14-16 |
| 18 | Conclusion and recommendations | List the main implications of the findings and place these in the context of other relevant literature. If appropriate, offer recommendations for policy and practice. | Discussion, Page 16-17 |
| 19 | Funding | Provide details of funding source (if any) for the review, the role played by the funder (if any) and any conflicts of interests of the reviewers. | Declaration, page 17 |

**Table S2: Data extraction table of multisectoral actions in primary health care**

| **Study** | **Country** | **type** | **Aim** | **Multisectoral actions** | **Key findings and authors conclusions** |
| --- | --- | --- | --- | --- | --- |
| Ramírez et al | South America | Review | Implementation of PHC | Community participation, intersectoral local planning and interdisciplinary team helped to implement CPHC  Inadequate coordination and participation challenging CPHC | - CPHC success factors were citizen's participation, intersectoral collaboration, optimization of intercultural care and interdisciplinary team, equitable geographical allocation of health teams, included focus of needed based and intercultural practices that focus on marginalized, participatory needs identification and prioritization, and intersectoral local planning of health actions and formulation of public health policy, and empowerment of local communities. - Challenges included Inadequate coordination between local, national, and international interests. Weak community participation, multidisciplinary and/or intersectoral action |
| Labonté et al | LMICs- India, Ethiopia, Kenya | Qualitative | To explore the strengths and weaknesses of CPHC initiatives at their local or national levels. | Removing walking distance to health posts -CHWs.  Awareness through ASHA.  Seeding funding for insurance funds. | - Health extension workers and volunteer female community health workers improved immunisation services, and family planning uses/HIV testing removing long walks to health posts in Ethiopia. - In India ASHA workers increased awareness among pregnant women. - Women groups supported seed fundings and developed insurance funds to assist pregnant women in DR Congo. In Kenya community health strategy improved coverage of child and maternal health services and safe access to safe drinking water |
| Álvarez-Bueno et al | Multiple | Systematic review | To evaluate the effectiveness of multifactorial interventions to decrease CVD | Multifactorial interventions improve the risk factors of CVDs | - Multifactorial community interventions are useful to improve cardiovascular risk factors levels the at-risk population. - SBP and SDP and cholesterol seem to be the CVD risk factors more likely to change |
| De Andrade et al | Latin America | Review | To explore the social determinants of health in Latin America | Social participation and intersectoral action enhanced equity | - Effectively used social participation with institutionalised deliberative mechanisms of participation (Brazil) and intersectoral action (Cuba), to enhance equity. - Achievements in population health and social outcomes expressed as country averages hide the unacceptably wide and persistent social and health inequities. |
| Evelyne de Leeuw | Multiple | Review | To review clarifies the conceptual foundations for integral health governance, policy, and action | Multisector contribute to health through governance, policy, research, and action including involvement of civil society, community perspectives, public private partnerships, and action | - Health is created largely outside the health sector. - Other sectors are called on to contribute to health through governance, policy, and action- political science, leadership studies, public health, empirical Health in All Policy research. - knowledge and evidence nexus approaches, and community perspectives, public and private sectors and with full involvement of civil society. |
| Fisher et al | Australia | review | To investigate intersectoral action on SDoH and health inequities in Australian health policy. | Intersectoral action in Australia concerned to individualised medical and behavioural interventions to client groups in other policy sectors. | - Health policy, strategies for intersectoral action were common but concerned with extending access to individualized medical or behavioural interventions to client groups in other policy sectors. - Strategies were mostly limited to addressing proximal factors, rather than policy settings affecting the distribution of socioeconomic resources. |
| van Eyk et al | Australia | Multi methods research | To present a five-year multi-methods research study of the SA HiAP approach and draws on data collected during interviews, observation, case studies, and document analysis. | Government agency readily understand health in all policies.  Health in all policies need to goal of facilitating joined up government in the process and address SDH through cross sectoral policy | - In South Australian health in all policy, government agencies readily understood HiAP as providing tools for improving the process of intersectoral policy development, while the more distal outcome-focused intent of improving equity was not well understood and gained less traction. - SA HiAP had dual goals of facilitating joined-up government for co-benefits (process focus); and addressing social determinants of health and inequities through cross-sectoral policy activity. |
| Maluka et al | Tanzania | Cross sectional | To reports on the design and implementation of service agreements between local governments and NSPS for the provision of primary health care services | Private sector’s involvement in services contract and services delivery | - The engagement of the government with NSPS are rooted in Tanzania’s long history of public-private partnerships in the health sector. - Delays in reimbursements, limited financial and technical capacity of local government authorities and lack of trust between the government and private partners affected the implementation of the contractual arrangements. |
| Javanparast et al | Australia | Cross sectional survey | To examine the strength and extent of collaborations between primary health care organisations and local government in population health planning | Australian regional organisation reported limited collaboration with local government | - Medicare Locals/ Primary Health Networks reported limited time and financial support for collaboration with local government. - Critical to collaborative planning included organisational capacity and resources, supportive governance and public health legislation mandating a role for local governments. |
| Assefa et al | Ethiopia | Country case | To explore feasibility and effectiveness of this approach in low-resource countries. | Multisectoral policies and strategies to achieve global policies | - Ethiopia has designed and implemented policies and strategies to guide its economic development, including poverty reduction strategies framed in the context of the MDGs and the SDGs. - Five-year development plans including multiple sectors are aimed at improving economic growth. |
| Jimenez Carrillo et al | El Salvador | Mixed methods | To explore stakeholders' perceptions related to the management of NCDs in PHC and the role of social participation | Emphasis intersectoral participation and interdisciplinary team including health promoters to address the NCDs communities | - The Salvadoran PHC and its comprehensive approach to NCDs with an emphasis on intersectoral participation has been positively perceived by stakeholders. - The interdisciplinary PHC-team brings holistic health care closer to the communities in which health promoters play a key role. |
| Kraef et al | Uganda | review | To explore the role of comprehensive primary health care to address the double burden of malnutrition. | multisectoral actions for nutrition- human rights, providers support in local multisectoral action, community empowerment for health diets and delivery of nutrition interventions | - bridging narratives and strengthening links between the PHC and the nutrition agenda with nutrition as a human right issue. - encouraging PHC providers to support local multisectoral action on nutrition. - empowering communities and patients to address unhealthy diets; and - ensuring the delivery of nutrition interventions. |
| Kriegel et al | Austria | Analytic Hierarch | To develop solution- and patient-oriented services that consider both the patients’ requirements as well as the medical, nursing, therapeutic and economic perspectives. | Social work is levers of primary health care which contribute to communication and cooperation of stakeholders | - The relevant levers being the professionalization of social work, competences of social work, communication, and cooperation of stakeholders. - Targeted development and optimization of the complex integration of social work into primary healthcare. |
| Tumusiime et al | WHO African Region | Discussion paper | To explain the role of multisector in pandemic, disasters and provide health services | Multisectoralism builds resilient health system through multisector actions have spill overs effects on health sector | - moving from fragmentation to integration, - working multisectorally/intersectorally, - ensuring implementation and knowledge exchange, and - rethinking resilience and embracing antifragility. Much of the health issues that ministries of health deal with are ‘spillovers’ from other sectors (e.g., Cholera needs to be addressed by water and sanitation, road traffic accidents to be addressed by transportation). |
| Bermejo et al | Cuba | Country case | To explain how intersectoral action to response COVID-19 in Cuba. | Single intersectoral plan helped to address COVID  Cuba’s National Health System has managed to COVIDguarantee an effective and equitable response | - The actions to face the pandemic began with preventive measures in the community, the preparation of a single national intersectoral government plan, continued in the isolation centers - the community with actions of surveillance and follow up of recovered patients. |
| DeHaven et al | USA | participatory research | To explain the role of community action to address social determinants of health | Social determinants of health can be addressed directly working with affected communities | - Training in high-risk communities for hundreds of doctors in training and demonstrated that the factors contributing to health - by working directly with and in affected communities to co-develop health care solutions across the broad range of causal factors and addressing the social determinants of health and population health. |
| Feryn et al | Multiple | review | To explain the role of social workers to implement these principles in primary health care settings. | Social workers have broad perspective on PHC | - Social workers’ broad perspective is valuable in PHC; however, their focus of social justice remains individual. - Strengthening of a social justice-based approach in primary health care, which is an issue that should be shared with other healthcare professionals. |
| Hazazi et al | Saudi Arabia | Qualitative | To examine the perspective of physicians on the current scope and content of NCDs management at PHCs including the contribution of the EHR system | Electronic medical records helped to documentation of NCDs patients | - The availability of the EHR helped organise their work and positively influenced NCDs patient encounters in their PHCs - emphasised the multiple benefits of EHR (e.g., efficiency, including the accuracy of patient documentation and the availability of patient information). |
| Nolan-Isles et al | Australia | Cross sectional | To investigate barriers and enablers to accessing healthcare services for Aboriginal people living in regional and remote Australia | Multisectoral strategies to provide health services to Aboriginal people – communication and coordination, trust cultural safety.Priorities health services and distance to health facility | - Improved coordination of healthcare services. - Better communication between services and patients. - Trust in services and cultural safety. - Importance of prioritizing health services by Aboriginal people. - Importance of reliable, affordable, and sustainable services. - Distance and transport availability. |
| Sitienei et al | Kenya | multiple case | To examine the implementation of community participation, through collaborative governance in primary health care facilities in Kenya, | Health committee can enhance multisectoral action  Poor participation, duplication, lack of clarity of responsibilities challenges the multisectoral action and collaborative governance. | - Committee members with the strongest influence and power had political connections or were retired government workers. - There was weakness in the inclusion of members of marginalized groups, and lack of clarity around processes involving stakeholders, the duplication of projects. - Lack of clarification of roles and responsibilities, conflict of interests, elaborate structures for stakeholder involvement, and variable competence of health facility committee members were the major challenges to enable collaborative governance. |
| Sturmberg et al | Multiple | Analytic study – qualitative | To explain how PHC can contribute to UHC | Top level health systems focus on polices and rules, regulations, and resources.  Bottom-up agency maintains focus on communities needs and limitations of system constraints | - Health systems are socially constructed organizational systems that are “functionally layered” in a hierarchical fashion - governments and/or funders at the top-level not only promote the goals of the system (policies) but also constrain the system (rules, regulations, resources) in its ability to deliver. - A need to focus on two key system features - political leadership and dynamic bottom-up agency that maintains everyone's focus on the goal and a limitation of system constraints so that communities can shape best adapted primary care services and truly meet the needs of their individuals, families, and community. |
| Super et al | Netherlands | Explorative | To study social innovation in sustainability and explain the multilevel perspective on transitions, | Intersectoral action and collaboration evolves through congruent processes at different levels in Rotterdam. | - intersectoral action between community organizations and policymakers evolves through congruent processes at different levels. - The multi-level perspective adds value to earlier approaches to research intersectoral collaboration for health promotion as it allows to better capture the politics involved in the social innovation processes. - transition processes involved in the development towards intersectoral action between youth care and sports, through a mixture of landscape developments, niche actors developing small-scale novel practices from the grassroots and change agency. |
| Tuangratananon et al | Thailand | mixed-method | To assess Thailand’s PHC capacity in providing NCDs services, identify enabling factors and challenges | Community engagement improves NCDs prevention.  Intersectoral actions can link the community and health promotion and address urbanisation and obesogenic environments | - Community engagement through village health volunteers improves NCDs awareness, supports enrolment in screening and raises adherence to interventions. - Crucial link between the health system and the community, are key in supporting health promotion and NCDs prevention and control. - Challenges- the dynamic of urbanization and socialization, especially living in obesogenic environments. |
| Madon et al | India | Qualitative | To interpret community health governance to improve understanding of how the government's policy vision | Village Health Sanitation and Nutrition Committees improved hygiene and sanitation | - Village Health Sanitation and Nutrition Committees focusing on sanitation, nutrition and hygiene which remain impediments to improving primary healthcare amongst poor and marginalized communities and - governance mechanisms of horizontal coordination, demand for accountability and self-help help to explain improvements. |
| Perveen et al | Multiple | Scoping review | To identify PHC principles are reflected in the implementation of national CHW programmes and how they may contribute to the outcomes of these programmes. | Intersectoral coordination was generally missing in the national community health worker’s programme | - Intersectoral coordination was generally missing in the national community health worker’s programme. - The cultural acceptability aspect of the principle of appropriateness was present in all programmes. |
| Rahimi et al | Iran | Qualitative | To investigate and identify the challenges affecting the performance of the primary health-care system in Iran. | Iran's PHC system improved through intersection actions especially on governance and human resources. | - Seven main themes consist of governance, manpower, resources, financial management, services delivery, trans‐sectional, and social and cultural. - The greatest challenges for Iran's primary health care (PHC) system are governance and human resources problems. |
| Holveck et al | Latin America and Caribbean region | Debate | To explain the contribution of multisector in neglected diseases marginalised populations. | Neglected diseases have vicious cycle with poverty and living conditions, this requires multisectoral actions | - Many of the neglected diseases do not directly cause high rates of mortality but contribute to an enormous rate of morbidity and a drastic reduction in income for the most poverty-stricken families and communities. - The persistence of this "vicious cycle" between poverty and poor health demonstrates the importance of linking the activities of the health sector with those of other sectors such as education, housing, water and sanitation, labor, public works, transportation, agriculture, industry, and economic development. |
| Adeleye et al | Multiple | Review | To review of non-health sector contribution | Intersectoral collaboration is strategic inputs of PHC | - The success of primary health care is rooted in weak strategic inputs, including intersectoral collaboration. Some encouraging evidence from programmes, projects, and studies suggests that intersectoral collaboration is feasible and useful. - The health sector expects inputs from other sectors which may not necessarily subscribe to a shared responsibility for health improvement, whereas the public expects “health” from the health sector. - The sector is challenged to mobilise all stakeholders for intersectoral collaboration through advocacy and programming. |
| Shankardass et al | Multiple | Scoping review | To identify and describe scholarly and grey literature. | Intersectoral approaches are complex | - The description of intersectoral approaches is complex. - multi-actor processes in the published documents were generally superficial and sometimes entirely absent. |
| Spiegel et al | Cuba | Qualitative | To consider how Cuba's acknowledged achievement of excellent health outcomes | Systematic and regular engagement of multisector and agencies was supportive to address health determinants | - Regular engagement of different sectors and other agencies in addressing health determinants was quite systematic and comparable in both municipalities. - Specific policies and organizational structures in support of intersectoral actions were frequently cited and illustrated in case scenarios. |
| Ndumbe-Eyoh et al | Multiple | Rapid systematic review | Case studies of intersectoral action are available, the impact of intersectoral action on the social determinants of health and health equity. | High (mid and upstream) interventions mixed, and downstream interventions had moderate effect in increasing the availability and use of services.  Collaboration of public and other sector created supportive environment for services | - The impact of upstream and midstream interventions showed mixed effects while downstream interventions were generally moderately effective in increasing the availability and use of services by marginalized communities. - Collaborations between public health and other sectors show promise in creating supportive environments and enhancing access to services for marginalized populations. |
| Rudolph et al | Multiple | Discussion paper | To review of role of health in all policies | Health in all policies incorporate collaborative decision makings  Health outcomes are largely depending on determinants outside the health thus require intersectoral collaboration | - Health in All Policies is an approach to improving the health of all people by incorporating health considerations into collaborative decision-making across sectors and policy areas. - Health in all approach believes good health is fundamental for a strong economy and vibrant society, and that health outcomes are largely dependent on the social determinants of health, - shaped primarily by decisions outside of the health sector and requires intersectoral collaboration as well as changes in government organizational structures and processes. |
| Anaf et al | Australia | Qualitative | To examine case studies of good practice in intersectoral action for health as one part of evaluating comprehensive primary health care | The value of intersectoral action for health and the importance of partnerships to primary health care services. | - The value of intersectoral action for health and the importance of partner relationships to primary health care services. - Facilitators of intersectoral action included sufficient human and financial resources, diverse backgrounds and skills and the personal rewards that sustain commitment while constraints were financial and time limitations, and a political and policy context. |
| Souza et al | Brazil | Descriptive | Analyzing public health practices, from the perspective of Family Health Strategy workers. | Challenges of multisector actions are little communication and articulation between the sectors | - Municipal management for intersectoral actions shows a lack of planning and faces challenges; and there is little communication and articulation between the sectors. - Intersectoral actions targeted at social health determinants, a demand inherent to the possibilities of advancing in the reduction of social and health-related inequalities. |
| Souza et al | Latin-American and Caribbean | integrative review | To identify the evidence about the repercussion of intersectoral programs / actions / strategies in the reduction of social inequities | Intersectional actions can improve the social determinants of health, living conditions and quality of life | - Intersectoral actions resulted in improved access to health, improved child nutrition indicators, better mental health care, the adoption of a healthy lifestyle, and improved quality of life. - The development and lives of children and adolescents are assigned to intersectoral actions. |
| Chaudhary et al | Nepal | Qualitative | To review the post-disaster health response provided by the MoH with support from the development partners and civil society | Post-disaster needs assessment, the response for ASRH services | - The establishment of female-friendly spaces near health facilities to offer a multisectoral response to gender-based violence - the setting up of adolescent-friendly service corners in outreach RH camps, t - The development of a menstrual health and hygiene management programme and the linkages established between adolescent-friendly information corners of schools and adolescent-friendly service centres in health facilities |
| Dhimal et al | Nepal | Qualitative | To gain the perspectives of key stakeholders involved in the Nepal MSAP on the barriers and facilitators to its implementation, through the participation of relevant sectors in the plan. | Working in sectors involved in the MSAP and line ministries including Office of the Prime Minister and Council of Ministries; academia; and professional organization. | - Multisector stakeholders with the MSAP, identifying a lack of leadership and poor dissemination. - Political and systemic transformation, since the adoption of the MSAP, was barrier to implementation. - International commitments to develop multisectoral action made by the Government of Nepal were identified as drivers. - The recent establishment of a separate section for NCDs and Mental Health within the Department of Health Services of MOHP and the promotion of a HiAP approach in recent national documents, were both considered to support implementation. |
| Ruducha et al | Nepal | mixed methods | To examine the structure and working relationships of the organizations that contributed to the development first MSNP with a focus on the role of partnerships | National multisectoral action plan for prevention and control of malnutrition awareness of nutrition, awareness of the MSAP, and barriers and facilitators to participation in the MSAP | - The development of the MSNP was related to the high density of organizational connections; the leadership role of the Nepal's National Planning Commission and the National Nutrition and Food Security Secretariat; and the bridging roles played by a few government ministries and development agencies. - Three types of working relationships: policy dialogue, strategic planning, and implementation. - Partners were less connected on MSNP implementation than for policy dialogue and strategic planning, which may have constrained collaborative scale-up efforts. - The Ministry of Agricultural Development, in particular, was the conduit for connecting non-health sectors into the broader network. |
| Mondal et al | India | Qualitative | To understand the processes involved in policy formulation and adoption, describing context, enablers, and key drivers, as well as highlight the challenges of policy. | Institutional architecture, political engagement, and legal interventions | - national law in India framed national policy, the signing of a global treaty, and the adoption of a dedicated national program. - The national Ministry of Health and Family Welfare, State Health Departments, technical support organizations, research organizations, non-governmental bodies, citizenry, and media, engaged in collaborative and, at times, overlapping roles. - Lobbying groups from the tobacco industry, were strong opponents with negative implications for policy adoption. - The state-level implementation relied on creating an enabling politico-administrative framework and providing institutional structure and resources to take concrete action. - The collaborative governance process were institutional mechanisms for collaboration, multi-level, and effective cross-sectoral leadership, as well as political prioritization and social mobilization. |
| Salunke | India | Qualitative | To engage multiple sectors, partners can leverage knowledge, expertise, reach, and resources, benefiting from their combined and varied strengths towards health outcomes. | MSAs refers to deliberate collaboration among various stakeholder groups and sectors to jointly achieve a policy outcome | - MSA help in addressing identified health issues in focused way as it helps in pooling the resources and formulating the common objectives. - Optimization of usage of resources by avoiding duplication of inputs and activities which tremendously improve program effectiveness and efficiency. - Willingness at the leadership and mandate at the policy level are necessary to plan and execute the successful multisectoral coordination. - All the major stakeholders require to share the common vision and perspective. - Developing institutional mechanism is utmost requirement as it will standardize the processes of intersectoral coordination. |

**Table S3: Cases studies of multisectoral actions in primary health care**

|  | **Macro-level: India’s tobacco control program** | **Macro-level: Nepal Multisectoral actions for nutrition program** | **Meso-level: Cuba’s Dengue Prevention Programme** | **Micro-level: NCDs prevention in Thailand** |
| --- | --- | --- | --- | --- |
| **Context** | - India’s tobacco consumption has long ranked among the highest in the world. Global Adult Tobacco Survey (GATS), 42.4% of men and 14.2% of women use tobacco. - Tobacco is a leading risk factor for major non-communicable diseases | - Undernutrition is the major public health problem in Nepal - Nepal joined scale up nutrition initiative in 2011 and approved the first 5-year Multisectoral Nutrition Plan (MSNP) in 2012, covering 2013–2017 | - Cuba’s Dengue Prevention Programme and Eradication of Aedes aegypti is a comprehensive set of intersectoral interventions. | - NCDs accounted for 74% of total mortality, with leading causes of cardiovascular diseases (23%), cancers (18%), chronic respiratory diseases (6%), and diabetes (4%). |
| **Objective of MSAs** | - To present an analysis of a collaborative and multisectoral policy | - To strengthen the multi-sector efforts of the National Planning Commission (NPC) and other stakeholders to enhance capacity development for improved nutrition at all levels of society in Nepal | - To eliminate and control of Aedes aegypti mosquito through environmental sanitation, hygiene, and collective household actions. | - To assess PHC capacities in managing NCDs, identify enabling factors and challenges and recommendations for improvement. |
| **Main actors, and composition of MSAs** | - Government ministry, technical support organisations, research organisations, NGOs, Media, policy entrepreneurs, individuals/citizenry | - Government Ministries (agriculture, health, education, local development, urban development, NPC), UN agencies, INGOs, Bilateral organisations. | - Health, communication, family doctors, water resource management experts, environmentalists | - Health care providers, community, services users |
| **Underpinning principles** | - The tobacco control issue, characterized by the need for interdependency and interaction, depended on strong coordination amongst all players as a pre-condition. - Multisectoral policy, a whole-of- society approach or the engagement of a whole system approach rooted in realizing the need for a joint action is necessary to propel the collaborative process. | - Undernutrition is multi-sectoral issue and emphasized that addressing stunting was beyond the capacity of a single ministry. - Integration of nutrition-specific and nutrition-sensitive interventions into existing development programmes. | - Intersectoral coordination and partnership at the operational level | - Multisectoral actions on prevention of NCDs is important - Prevention is better than cure, but preventive and promotive actions require MSAs |
| **Contents of MSAs** | - Generation of policy alternatives- the role of research and evidence (sensitize for tobacco control) - Deliberations and Consultations: Discussions and Expert Opinions - The Mainstay: Political Sensitization and Legal Intervention - Tobacco Industry Lobbying to Influence Policy Decisions - Multi-level, Cross-sectoral Leadership - Institutional Mechanisms for Collaboration - Political Attention and Mobilization | - Working relationships of agencies including multiorganizational collaboration and intensity of relationship - Policy dialogue and development - Strategic planning - Scale up implementation | - Underpinned by legislation, the programme includes the local government, the Ministry of Public Health, community associations, family doctors, water resources management, the anti-mosquito brigade, and several civil society organisations. - the programme has led to a reduction of dengue infections and improved environmental management for vector control. - The Dengue Prevention Programme’s grounding at the primary health-care level, with implementation leadership provided by provincial and municipal governments and participatory approaches to create local level needs assessment and action plans, offers important lessons for intersectoral action for universal health coverage, and sustainable development. | - Community engagement through village health volunteers improves NCDs awareness, supports enrolment in screening and raises adherence to interventions. Village health volunteers, the crucial link between the health system and the community, are key in supporting health promotion and NCDs prevention and control. - Collaboration between provincial and district hospitals in providing resources and technical support enhance the capacity of PHC centres to provide NCDs services. |
| **Outcome** | - Collaborative action, mobilization of stakeholders of legal and political, and social advocacy to bring about intended policy change. | - The agriculture sector can play a larger bridging role in mobilizing non-health sectors to participate in broader multisectoral networks for nutrition. - The development of Nepal's Multisectoral Nutrition Plan led to establish a strong governance structure at the national level and fostered a high level of organizational connectivity between sectors and stakeholders. | - The experience of Cuba highlights the importance of investment in health promotion and disease prevention as integral components of UHC. | - Empower individuals and citizens to optimize their health, particularly in urban contexts. - Health literacy at the heart of NCDs prevention and control and address commercial determinants of health. - The PHC approach has limited capacity concerning multi-sectoral collaboration to address the SDH. |
