# Supplementary material for: Multisectoral actions in primary health care: A realist synthesis of scoping review
Source: PLoS One. 2023 Aug 10;18(8):e0289816. doi: 10.1371/journal.pone.0289816 (PMC10414560; doi:10.1371/journal.pone.0289816)
Supplement: Attachment — Submitted filename: Point by point response_.docx [file pone.0289816.s003.docx]

Point by point to the editor's and reviewers’ comments

The authors’ team would like to thank you for constructive feedback on our manuscript. We appreciate your insightful and constructive feedback. We fully agree with your views. We have revised as suggested. In this document, we have responded point-by-point on your feedback, and clarification of the concerns where necessary.

Additional Editor Comments:

| Comments | Response |
| --- | --- |
| Title – the changes on the last part of the title to me did not look great. Up to the authors but the previous line saying a realist synthesis of scoping review looked to be a better presentation of the work rather than the new one changed in the revised version. | Corrected as suggested. |
| Intro –In the starting line of the introduction, if you could interweave intersectoral coordination on health referring to alma ata into what you have written as MSA being fundamental principle of PHC, it would make this read even more beautiful.  In line 98, the use of sentence We employed….. gives a sense of methods section being injected in the intro. Authors might want to reconsider if they really want to position any methods language in the intro. | Thank you for this feedback. We revised the concept of intersectional coordination in 1978. We hope this make sense to the readers.  We revised the method-related contents from the introduction section. |
| One more serious and thorough copyediting required. Abbreviations – there still remain issues. Like MSA coined early in the abstract but later in couple of places again full form is still there (and in few places in the main body of the manuscript after it has been abbreviated in the beginning).  It is suggested to take this manuscript through a thorough copyediting to make sure these kind of errors as well as errors like inconsistent use of capital letters to name a few are corrected including all other typographical issues. Some more examples of typographical issues (there could be more) Line 106 - …different categories MSAs on health…. Is something missing here? Probably ‘of’? Line 470 – did the authors mean multilevel context of health systems and not health and systems. | We checked thoroughly these issues and corrected.  We edited our manuscript throughout. Thank you so much for suggestions. |

Thank you so much for so constructive feedback on our work.
